# Determination of Opium Alkaloid Content in Poppy Seeds Using Liquid Chromatography Coupled with a Mass Spectrometer with a Time-of-Flight Analyzer (UPLC-TOF-HRMS)

**DOI:** 10.3390/foods13172826

**Published:** 2024-09-05

**Authors:** Agnieszka Zapaśnik, Adam Pierzgalski, Marcin Bryła

**Affiliations:** 1 Department of Microbiology, Waclaw Dabrowski Institute of Agricultural and Food Biotechnology—State Research Institute, Rakowiecka 36, 02-532 Warsaw, Poland; 2 Department of Food Safety and Chemical Analysis, Waclaw Dabrowski Institute of Agricultural and Food Biotechnology—State Research Institute, Rakowiecka 36, 02-532 Warsaw, Polandmarcin.bryla@ibprs.pl (M.B.)

**Keywords:** morphine, opium alkaloids, codeine, poppy seeds, food safety, liquid chromatography

## Abstract

Opium poppy is a plant used in both the pharmaceutical and food industries. Substances found on the surface of dry poppy seeds belong to the group of opium alkaloids. However, the presence of these substances in food products poses a risk to consumer health, which is why new permissible levels for both substances in poppy seeds and derivative products have been introduced in Regulation (EU) 2023/915. This research aimed to analyze the content of all six opium alkaloids in poppy seeds provided directly by producers as well as those available on the local market in Poland. The research confirmed the presence of morphine in all examined poppy seed samples. The alkaloid content ranged from 12.46 to 102.86 mg/kg for seeds purchased in local markets and from 1.1 to 110.1 mg/kg for seeds obtained directly from producers. Both groups showed similar levels of morphine content as well as other OAs, which significantly exceeded the permissible limit of 20 mg/kg set by the European Commission (EU) 2023/915. These results indicate that the presence of morphine and other opium alkaloids in poppy seeds exceeds permissible levels, posing a serious health issue and necessitating further research and improvement in processing methods.

## 1. Introduction

Opium poppy (*Papaver somniferum* L.) is well-known in medicine due to the presence of medically important metabolites in its seeds. According to the literature data, the genus consists of about 149 species occurring in the subtropical and temperate regions of the Northern Hemisphere [1,2]. The substances mentioned above demonstrate pharmacological activity, such as inhibition of mucus secretion in the lungs, but most of all, an analgesic effect [3,4]. Among several metabolites, the most important are morphine and codeine, which are present on the surface of the poppy seeds in the proper phase of growth [5].

However, not all genotypes of *Papaver somniferum* L. demonstrate the ability to biosynthesize opium alkaloids (OAs). The species with low alkaloid content are useful in the food industry for oil or seed production. The cultivation of opium poppy is illegal or limited by specific legislative regulations in most parts of the world [1]. Currently, the list of countries where the cropping of *Papaver somniferum* L. is legal for the food and pharmaceutical industry is strictly defined, consisting of Turkey, the Czech Republic, Slovakia, France, Hungary, Poland, Russia, Spain, Romania, Holland, Australia, India, Iran, Canada, and Central and Southern America [2]. In some countries (the Czech Republic), the low-morphine varieties are cultivated only for the use of the food industry; however, in other regions, the opium poppy can be cultivated for both culinary and pharmaceutical uses, including France, Spain, or Australia [2].

The poppy seeds do not contain OAs themselves, but they become contaminated with the plant-synthesized latex (milky sap), which is rich in OAs such as morphine, codeine, thebaine, papaverine, and noscapine [6]. Contamination occurs due to insect damage or improper harvesting practices. Recent studies have proved the presence of OAs in poppy seeds, in many cases in disturbingly high concentration and highly widespread due to the heterogeneous contamination [7,8,9]. According to the EFSA Scientific Opinion, morphine is the major alkaloid occurring in poppy seeds. Furthermore, morphine and codeine have a high level of co-occurrence, the same as thebaine and codeine or thebaine and morphine [10]. The European Commission requested that the EFSA update the Scientific Opinion on OA safety. The CONTAM Panel (Panel on Contaminants in the Food Chain) estimated the acute reference dose (ARfD) of 10 μg of morphine/kg body weight (bw). In the case of codeine, converting codeine to morphine equivalents using a factor of 0.2 was recommended [10]. According to the newest Commission Regulation (EU) 2023/915, the highest content of morphine (the sum of morphine and codeine × 0.2 factor) in whole, shredded, and ground poppy seeds should not exceed 20 mg/kg [11]. Morphine is mostly absorbed in the gastrointestinal (GI) tract and then distributed throughout the whole organism [12]. Though the activity of morphine primarily occurs in the brain, the main alkaloid or its metabolites may diffuse into the placenta and be present in milk [13]. OAs essentially affect the central nervous system (CNS), but according to current knowledge, the cardiovascular and respiratory systems may also be affected [14,15]. Due to the factors mentioned above, OAs may cause serious health problems, especially in infants, children, the elderly, and people with poor medical conditions.

These days, the most common methods of the determination of OAs in poppy seeds are gas chromatography coupled with mass spectrometry (GC-MS) or liquid chromatography coupled with tandem mass spectrometry (LC-MS/MS) [16]. Most commonly, the limits of detection are below 1 mg/kg for morphine and other opium alkaloids.

To meet new legal regulations in the EU regarding the maximum permissible content of morphine in poppy seeds, as well as the need to conduct a risk assessment related to the intake of other opium alkaloids with food, the aim of the research was to develop a method for the simultaneous determination of six opium alkaloids (including morphine and codeine) in poppy seeds. Using the developed method, an assessment of the contamination of poppy seeds in retail trade, as well as uncleaned poppy seeds intended for consumption purposes with these compounds, was carried out.

## 2. Materials and Methods

### 2.1. Chemical and Reagents

Water (LC-MS purity) and methanol (HPLC and LC-MS purity) were provided by Chemsolve (Łódź, Poland). Deionized water was purchased from Hydrolab, Straszyn, Poland. Formic acid (LC-MS purity) was purchased from Chemlab (Zedelgem, Belgium). Ammonium carbonate was provided by Sigma-Aldrich (Darmstadt, Germany).

### 2.2. Research Material

A total of 6 samples of commercially available poppy seeds were purchased in local supermarkets in Warsaw (2 samples of white poppy seeds, 2 samples of blue poppy seeds, and 2 samples of black poppy seeds). Additionally, 59 samples of poppy seeds were obtained from poppy producers before marketing authorization.

### 2.3. Sample Preparation (OAs Measurement)

Poppy seeds (10 g) were weighed in a conical flask (250 mL), and then 100 mL of the solution of methanol with 0.1% formic acid was added. The samples were homogenized in the Unidrive X 1000 homogenizer (CAT Scientific, Inc., Pase Robles, CA, USA) for 1 min. After homogenization, the supernatant was poured into a new flask, and then the residue of poppy seeds was mixed with 100 mL of the solution (methanol with 0.1% formic acid) for the second time. The procedure of homogenization was repeated. The next step was a combination of two extracts and filtration with the usage of a folded filter paper (PureLand, Stargard, Poland). Then, 0.1 mL of filtrate was transferred into 10 mL volumetric flask and filled to the mark with methanol. After filtering the supernatant through a syringe filter with a pore size of 0.2 μm, the solution was analyzed using liquid chromatography coupled with a mass spectrometer.

### 2.4. LC/MS Analysis

The content of OAs in poppy seeds was determined by An H-class liquid chromatograph coupled with a mass spectrometer with a time-of-flight analyzer (UPLC-TOF-HRMS; Waters, Milford, MA, USA). Analytes were separated on a 2.1 × 100 mm, 1.6 µm UPLC C18 Cortecs chromatographic column (Waters, Milford, MA, USA) with an appropriate pre-column, operated with a gradient regime. The mobile phases consisted of water containing 10 mM ammonium carbonate (phase A) and methanol (phase B). The flow rate was 0.2 mL/min, and the column temperature was 40 °C. The following gradient was used: 55% phase A from 0 to 3 min; 8% phase A from 3 to 11 min; 55% phase A from 11 to 15 min. A total of 1 µL of sample was injected into the column. The mass spectrometer was operated in positive polarization with electrospray ionization (ESI). The ion source and desolvation temperatures were 120 °C and 370 °C, respectively. The flow rate of the atomizing gas (nitrogen) was 750 L/min, and the flow rate of the drying gas was 30 L/min. The voltage on the capillary was 3000 V. The V mode of ion optics was used. The mass spectrometer was calibrated using a standard leucine–enkephalin solution. The test compounds in the cereal samples were identified by comparing their molecular masses and retention times to the standards of these substances. For quantitative analysis, the following molecular ions (*m*/*z*) were used for the individual analytes: morphine—286.15 (M + H)+; codeine—300.18 (M + H)+; oripavine—298.15 (M + H)+; papaverine—340.15 (M + H)+; thebaine—312.15 (M + H)+; noscapine—414.25 (M + H)+.

### 2.5. Method Validation

Due to the lack of detailed guidelines regarding the requirements of the analytical methods of analysis of opium alkaloids, the validation was based on the requirements for quality control analytical and validation of pesticide residue analysis methods described in the documents SANTE/12682/2019 (method recovery of 70–120% with a precision of at most 20%) [17]. In the validation experiment, the measurement range covering each analyte was determined with the range of the calibration curve, the limit of detection and quantification (LOD and LOQ) (concentrations at which the signal-to-noise ratio (S/N) is at least 3, respectively, and 10), method recovery (R%), and precision value (expressed as relative standard deviation RSD%). Calibration curves were prepared based on the analysis of calibration solutions. Based on a comparison of the slope coefficients of these curves (SSE = slope coefficient of the curve prepared in matrix solutions/slope coefficient of the curve prepared in pure solvent × 100), the matrix effect was determined, where it was assumed that if the SSE value is 95–105%, no matrix effect is observed. As the value obtained met the above criteria, the calibration curve was prepared using pure methanol. As the poppy used in the validation experiment was characterized by the presence of alkaloids and to eliminate the effect of material heterogeneity, recoveries were determined by enriching the poppy methanolic extract at 0.25 mg/kg, 2.5 mg/kg, 7.5 mg/kg, and 22.5 mg/kg of each opium alkaloid (*n* = 6).

### 2.6. Data Analysis

The concentration analyses of individual samples were performed in triplicates. Data analysis involved calculating the means, medians, and standard deviations using Microsoft Excel 365 to illustrate the reproducibility and reliability of the results.

## 3. Results and Discussion

### 3.1. The Method’s Evaluation

Determination coefficients (R^2^) for all matrix-fit curves obtained were above 0.98. The range calibration curve and coefficient of linear determination (R^2^) for individual analytes are included in Table 1. The determined LOD (S/N = 3) and LOQ (S/N = 10) values were 0.06 and 0.2 mg/kg for each test compound, respectively.

According to the adopted criteria, the method was considered compliant when the repeatability (RSDr) was 20% or less and the recovery (R%) was 70–120%. In all cases, the results of the validation experiment met the required criteria (Table 2).

Before the main experiment, the extraction method was selected based on the analysis of naturally contaminated alkaloids in ground poppy seeds. For this purpose, four extractions with an acidic solution were carried out with methanol, and the percentage of analyte was determined at individual extraction stages. The experiment mentioned above proved that a double extraction would be an appropriate approach.

### 3.2. The Concentration of OAs in Poppy Seeds

**Figure 1:**
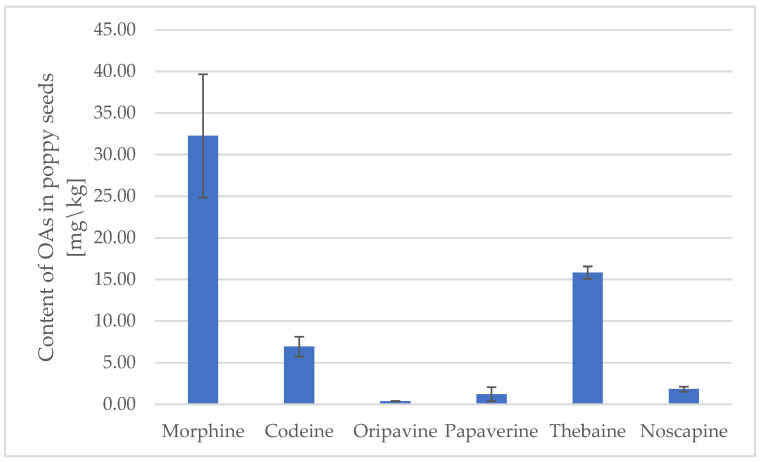
The average content of OAs in commercially available poppy series [mg/kg].

**Figure 2:**
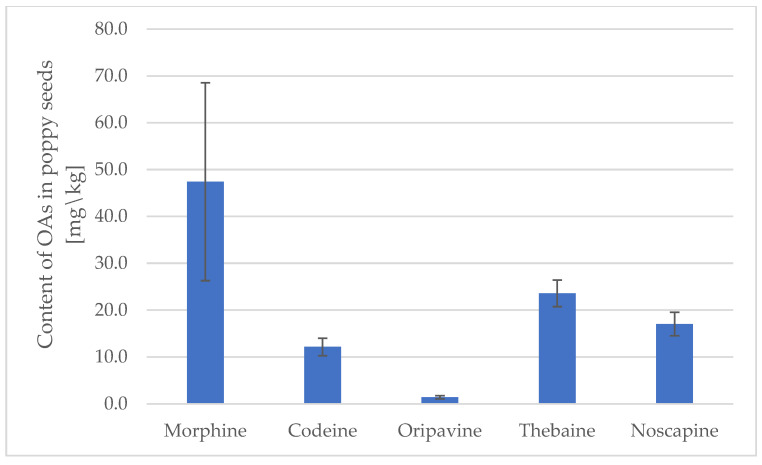
The average content of OAs in poppy seeds from local producers [mg/kg].

There is a wide spectrum of colors of poppy seeds, from white and blue to black and yellow. Black and blue seeds are most popular in the US or Europe, while yellow and white are used in South Asia, especially in Indian cuisine. All of them come from multiple genotypes of *P. somniferum*, commonly named poppy plant [18]. To estimate the concentration of OAs in poppy seeds, the black or blue variety with only two probes of white poppy seeds was taken for the experiment. The content of all six OAs are provided in figures [Figure 1 and Figure 2] and Table 3.

Among the average content of studied opium alkaloids (OAs), morphine was present at the highest concentration, ranging from about 30 to 45 mg/kg, depending on the type of poppy seeds [Figure 1 and Figure 2]. Thebaine was also found at a relatively high level of 15–23 mg/kg in both store-bought and locally sourced poppy seed samples. The content of codeine varied between approximately 5 and 10 mg/kg. Papaverine and oripavine contaminated the seeds to a minor extent, whereas noscapine was present at a level of 15 mg/kg in seeds provided by local producers.

This study confirmed the presence of morphine in all examined poppy seed samples. The alkaloid content ranged from 12.46 to 102.86 mg/kg for poppy seeds purchased in local markets and from 1.1 to 110.1 mg/kg for seeds obtained directly from producers. Comparing the results, it was found that the morphine content in samples from both groups was at similar levels. Considering the latest guidelines regarding the maximum permissible morphine content in poppy seeds set by Commission Regulation (EU) 2023/915 (20 mg/kg), the presence of this alkaloid in individual samples is significantly higher than the allowable limit [11]. This is particularly concerning, given that morphine is not the only alkaloid found at high levels, as will be discussed in subsequent paragraphs.

Similar results were obtained by other researchers who analyzed poppy seeds for the presence of OAs. Casado-Hidalgo et al. [8] confirmed the presence of morphine in poppy seed samples at levels ranging from 30.55 to 57.94 mg/kg. Other authors also demonstrated high levels of morphine, ranging from 0.2 to 241 mg/kg [19] and from 3.6 to 261 mg/kg [4]. In the studies conducted by Carlin et al. [7], morphine content was relatively low, ranging from 0.9 to 12.58 mg/kg in poppy seed samples.

Zentai et al. [20] analyzed the presence of morphine in commercially available poppy seeds in Hungary from 2001 to 2010. Morphine was present in almost all samples during the study period, with the highest concentration reaching 533 mg/kg. Their studies also estimated the daily exposure risk and found that, since poppy seeds are usually used as a flavoring addition to bakery products, the exposure to morphine is relatively low, ranging from 25.6 to 34.7 µg/kg bw per day, depending on the study period.

Codeine was present in all examined samples from both study groups at levels ranging from 0.25 to 37.05 mg/kg (market-purchased) and 1.3 to 24.3 mg/kg (local producers). It was found that the alkaloid content in store-bought samples was slightly higher compared to those from producers. Here too, attention should be paid to the exceeded permissible limit set by Commission Regulation (EU) 2023/915 [11]. Slightly lower concentrations were reported in poppy seeds by Carlin et al. [7] and Casado-Hidalgo et al. [8], with ranges of 0.39 to 6.14 mg/kg and 4.46 to 10.88 mg/kg, respectively. Lopez et al. [19] confirmed the presence of codeine at higher levels in poppy seeds, ranging from <0.1 to 348 mg/kg.

Oripavine shows similar effects to morphine, but the toxicological and occurrence data are insufficient to estimate a risk assessment [10]. Therefore, further research is needed at various stages of food production processing, as well as testing the toxic potential of other opium alkaloids in poppy seeds to enable comprehensive hazard characterization [13]. The presence of oripavine was confirmed in two out of six poppy seed samples purchased in stores at low levels ranging between 0.37 and 0.39 mg/kg and in 36 out of 59 samples provided by producers at levels <LOQ to 5.7 mg/kg. Casado-Hidalgo et al. [8] reported slightly higher values of oripavine in poppy seeds (5.3 to 9.1 mg/kg).

Papaverine and noscapine are characterized by less toxicity compared to morphine based on the limited data, and exposure to these substances is lower [10]. The presence of papaverine was confirmed in four out of six store-bought poppy seed samples, in trace amounts ranging from <LOQ to 4.21 mg/kg. Papaverine was not detected in poppy seeds from local producers. However, it should be noted that papaverine can be present in relatively high amounts, as confirmed by other authors. Casado-Hidalgo et al. [8] found this alkaloid at levels ranging from 5.17 to 12.06 mg/kg, while Lopez et al. [19] reported papaverine in poppy seeds at levels ranging from <0.1 to 3.8 mg/kg. The presence of noscapine was confirmed in all examined samples from both groups at levels ranging from 0.31 to 4.54 mg/kg for store samples and 0.4 to 29.4 mg/kg for producer samples. Similar values were reported by other authors, with ranges of 8.0 to 39.7 mg/kg [8], 0.3 to 6.83 mg/kg [7], and 0.1 to 6.0 mg/kg [19].

To date, morphine or codeine constitute the main concerns in the risk assessment of opium alkaloids (OAs) in poppy seeds, but limited data indicate higher acute toxicity of thebaine compared to morphine [21]. Thebaine was present in all examined poppy seed samples, both market-purchased and producer-provided, at levels ranging from <LOQ to 32.67 mg/kg and 1.1 to 68.8 mg/kg, respectively. Samples provided by local producers were more contaminated with thebaine compared to those from store shelves. Casado-Hidalgo et al. [8] confirmed the presence of thebaine in poppy seeds at levels ranging from 7.59 to 21.03 mg/kg. Similarly, high values were reported by Carlin et al. [7], with levels ranging from 0.37 to 37.22 mg/kg. In the studies by Lopez et al. [19], thebaine was found at even higher levels, ranging from <0.1 to 106 mg/kg, compared to the present study and other authors.

### 3.3. Methods of Reduction of OA Contamination on Poppy Seed Surfaces

To date, only a limited number of studies have investigated the changes in opium alkaloid (OA) levels during different kinds of pre-treatments and processing, focusing on morphine and, to a lesser extent, codeine. Furthermore, the results vary depending on the food matrix (such as muffins, buns, cakes, or bread), thermal processing, and heating parameters, ranging from nearly complete degradation of OAs [22] to minimal degradation [4]. These discrepancies can also be attributed to the high variability in OAs content within different portions of the same batch [23]. Based on the recommendations of Commission Regulation 2014/662/EU, pre-treatment and processing methods should be implemented to decrease the content of OAs on the surface of poppy seeds [24]. These methods include washing or soaking in water, thermal treatment (with higher temperatures providing greater reduction efficiency), and grinding.

Casado-Hidalgo et al. [25] analyzed the effectiveness of the fermentation process on the degradation of OAs and their presence in dairy-fermented beverages enriched with poppy seeds. They confirmed the presence of morphine, papaverine, and noscapine in all tested samples, although below the limit of quantification (LOQ). Furthermore, they found that fermentation could reduce OAs by 33–80% within the first few hours of the process. Lactic acid bacteria are well known for their antimicrobial and degradation properties regarding various food contaminants, such as mycotoxins. Their effectiveness is due to the production of metabolites like organic acids or bacteriocins, as well as their bioabsorption capabilities [26]. In the case of opium alkaloids, their degradation may involve enzymatic activity from these bacteria, including proteases, glycosidases, and esterases, which can potentially lead to the breakdown of alkaloids. Additionally, the presence of acids (e.g., lactic acid) and the resulting low pH may influence the hydrolysis of these compounds. These results encourage further exploration to develop the most beneficial process parameters and determine which strains of LAB most effectively contribute to OAs reduction. Shetge et al. [4] indicated that the baking process alone is insufficient to achieve a significant reduction in OAs in poppy seeds. They found that to enhance the degradation of morphine, codeine, and thebaine, poppy seeds require extended thermal processing at high temperatures (200 °C) and/or pre-washing. The reduction achieved through high-temperature processing alone is approximately 50%, while the combination of washing and thermal processing reduces OAs content by about 50–80%. Vera-Baquero et al. [23] investigated the thermal degradation of opium alkaloids in breadsticks made with corn flour and containing *Papaver somniferum* L. seeds. A total of six opium alkaloids were analyzed, including morphine, codeine, thebaine, papaverine, and noscapine. The research found that baking at 180 °C for 20 min led to a substantial reduction in these alkaloids, with morphine and codeine showing up to 100% degradation. The degradation of thebaine, papaverine, and noscapine ranged from 14% to 58%. Additionally, their study revealed that the degradation of morphine and codeine was more pronounced when the seeds were used as a topping on the breadsticks. Furthermore, Kuntz et al. [13] indicated that morphine and codeine degradation is more pronounced when the seeds are used as a topping for bakery products. Since, e.g., the core temperature of the breadsticks only reached approximately 100 °C, the stability of the OAs in these samples is the result of inadequate heat exposure due to the matrix protection. Kleinmeier et al. [27], in their commentary, indicated that the crucial aspects in the thermal degradation of opium alkaloids are the activation energy (Ea) and half-lives of the primary opium alkaloids in poppy seeds. Their measurements revealed that morphine and codeine have relatively low thermal sensitivity. Specifically, their findings showed that the half-lives of these alkaloids at 200 °C were between 32 and 39 min.

In conclusion, the pre-treatment and cleaning methods described above are widely known and contribute to the reduction in OAs, including morphine, to some extent, but not completely. Further investigation into the degradation of alkaloids in poppy seeds is crucial. The reduction in opium alkaloids in poppy seeds plays a role in shaping safety and processing guidelines from government agencies [10]. For this reason, it is essential to carry out more experiments to ensure accurate and reliable recommendations.

## 4. Conclusions

This study successfully developed and validated a method for the simultaneous determination of six opium alkaloids (OAs) in poppy seeds using liquid chromatography coupled with mass spectrometry (LC-MS/MS). The method proved effective, meeting the required criteria for precision and accuracy.

The analysis revealed that morphine was the predominant alkaloid present in poppy seeds, with concentrations ranging from 12.46 to 102.86 mg/kg in commercially available seeds and 1.1 to 110.1 mg/kg in locally sourced seeds. Other OAs, including codeine, thebaine, oripavine, papaverine, and noscapine, were also detected at varying levels, often exceeding the permissible limits set by the European Commission.

The presence of these alkaloids, particularly morphine and thebaine, poses significant health risks, necessitating stringent regulatory oversight and the implementation of effective pre-treatment methods to reduce OA contamination. Washing, soaking, thermal treatment, and fermentation were identified as potential methods for reducing OA levels, with varying degrees of effectiveness.

Overall, this research highlights the need for continuous monitoring of OA levels in poppy seeds and the adoption of best practices in processing to ensure consumer safety. Further studies are recommended to optimize reduction methods and assess the toxicological impact of lesser-known alkaloids like oripavine.

## Figures and Tables

**Table 1:** Characterization of the method for the determination of opium alkaloids in poppy.

Analyte	Range Calibration Curve (ng/mL)	Range of Linearity (mg/kg)	Coefficient of Linear Determination (R^2^)
Morphine	0.63–160	0.13–32	0.9914
Codeine	0.63–160	0.13–32	0.9802
Oripavine	0.63–160	0.13–32	0.9942
Papaverynine	0.63–160	0.13–32	0.9880
Thebaine	0.63–160	0.13–32	0.9912
Noscapine	0.63–800	0.13–16	0.9840

**Table 2:** Determined recovery (R%) and precision (RSD%) values for different levels of opium alkaloids tested in poppy samples.

Analyte	Fortification Level *							
	0.25 mg/kg		2.5 mg/kg		7.5 mg/kg		22.5 mg/kg	
	R%	RSD%	R%	RSD%	R%	RSD%	R%	RSD%
Morphine	100	8.2	108	4.8	109	3.3	110	12.1
Codeine	99	14.1	115	3.7	115	1.8	114	12.0
Oripavine	94	11.7	108	12.5	91	16.3	90	18.0
Papaverine	93	18.8	110	2.2	113	2.1	117	8.7
Thebaine	107	11.0	107	9.1	103	3.5	101	11.5
Noscapine	95	19.1	116	4.5	117	2.8	120	9.8

* Number of independent repetitions *n* = 6.

**Table 3:** The content of OAs in commercially available poppy series and poppy seeds from local producers [mg/kg].

Commercially Available Poppy Series					Poppy Seeds from Local Producers			
	% Positive Samples	Average [mg/kg]	Median	Min–Max	% Positive Samples	Average [mg/kg]	Median	Min–Max
Morphine	6/6	32.26 ± 7.4	15.81	12.46–102.86	59/59	47.41 ± 21.13	46.7	1.1–110.1
Codeine	6/6	6.93 ± 1.19	2.36	0.25–37.05	59/59	12.16 ± 1.87	12.9	1.3–24.3
Oripavine	2/6	0.38 ± 0.01	0.38	0.37–0.39	36/59	1.41 ± 0.35	1.2	<LOQ *–5.7
Papaverine	4/6	1.22 ± 0.85	0.37	<LOQ *–7142	0/59	<LOQ *	<LOQ *	<LOQ *
Thebaine	3/6	15.82 ± 0.76	15.78	<LOQ *–32.67	59/59	23.57 ± 2.84	23.5	1.1–68.8
Noscapine	6/6	1.83 ± 0.30	1.88	0.31–4.54	59/59	17.05 ± 2.49	19.5	0.4–29.4

* <LOQ = 0.2.

## Data Availability

The original contributions presented in the study are included in the article, further inquiries can be directed to the corresponding author.
